# Modern Management of Localized Renal Cell Carcinoma— Is Ablation Part of the Equation?

**DOI:** 10.15586/jkcvhl.v9i3.233

**Published:** 2022-08-15

**Authors:** Zev Leopold, Rachel Passarelli, Mark Mikhail, Alexandra Tabakin, Kevin Chua, Ronald D. Ennis, John Nosher, Eric A. Singer

**Affiliations:** 1 Section of Urologic Oncology, Rutgers Cancer Institute of New Jersey and Rutgers Robert Wood Johnson Medical School, New Brunswick, NJ, USA;; 2 Department of Radiation Oncology, Rutgers Robert Wood Johnson Medical School, New Brunswick, NJ, USA;; 3 Department of Radiology, Rutgers Robert Wood Johnson Medical School, New Brunswick, NJ, USA

**Keywords:** cryoablation, microwave ablation, percutaneous ablation, radiofrequency ablation, renal cell carcinoma, stereotactic ablative body radiotherapy

## Abstract

While the gold-standard for management of localized renal cell carcinoma (RCC) is partial nephrectomy, recent ablative strategies are emerging as alternatives with comparable rates of complications and oncologic outcomes. Thermal ablation, in the form of radiofrequency ablation and cryoablation, is being increasingly accepted by professional societies, and is particularly recommended in patients with a significant comorbidity burden, renal impairment, old age, or in those unwilling to undergo surgery. Maturation of long-term oncologic outcomes has further allowed increased confidence in these management strategies. New and exciting ablation technologies such as microwave ablation, stereotactic body radiotherapy, and irreversible electroporation are emerging. In this article, we review the existing management options for localized RCC, with specific focus on the oncologic outcomes associated with the various ablation modalities.

## Introduction

### Treatment options for localized renal cell carcinoma

The incidence of renal cell carcinoma (RCC) has increased over the past four decades, with recent estimates predicting 79,000 new cases of kidney cancer in 2022 (1, 2). Stage 1 tumors, which are ≤7 cm and contained to the kidney, comprise 40–50% of new cases (1, 3). This rise is, in part, due to the improved detection of incidental renal tumors, with the advent of and widespread usage of cross-sectional imaging (1, 4).

The management of small renal masses (SRMs), defined as ≤ 4 cm or cT1a tumors, continues to evolve with our improved understanding of tumor biology and application of technology. Treatment options for SRMs include radical or partial nephrectomy (PN), renal mass ablation, and active surveil-lance (5–7). While radical nephrectomy (RN) was once the gold standard for most cases of localized RCC, a shift toward minimally invasive surgery and interest in nephron-sparing procedures led to the popularization of PN as standard of care for localized RCC (8). However, in select patients with localized RCC, focal ablative therapies and active surveillance have gained traction as possible alternatives (8).

**Figure S1:**
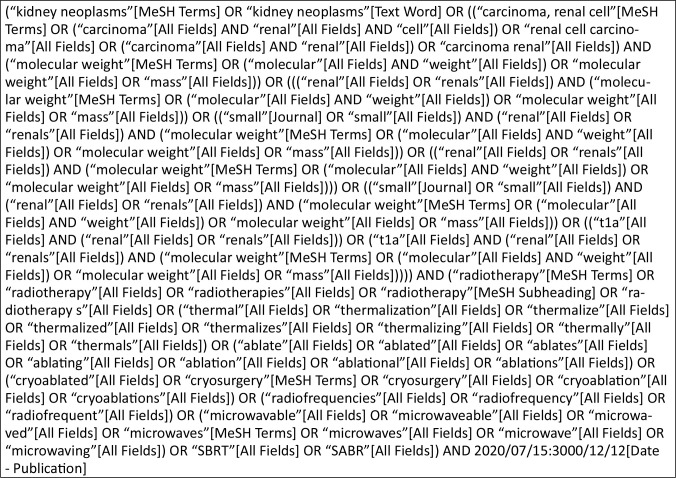
PubMed Search Query.

Although surgery for localized RCC is the most common treatment and has traditionally been associated with higher cancer-specific survival (CSS), emerging evidence has demonstrated comparable oncologic outcomes with ablative therapy (7, 9–11). Clinicians must engage in shared decision-making with their patients, considering the risks and possible complications paired with patient factors, their treatment goals, and institutional capabilities (5, 6). Review articles play a vital role in keeping patients and clinicians abreast to changing clinical paradigm, especially when considering topics such as the management of SRMs. The advent of numerous ablative modalities and their subsequent outcomes studies necessitates periodic, in-depth examination of the evidence. In this review, we discuss various ablative modalities for localized RCC, their outcomes, and how the current literature supports the integration of ablation into clinical practice. We initially conducted a broad literature review through PubMed (Supplemental Figure 1). Articles were further examined for inclusion in our review with particular attention paid to randomized clinical trials (RCTs). Ongoing trials and or those without published results were discovered through clinicaltrials.gov.

### Patient evaluation and selection

All patients with localized RCC should first undergo a detailed history and physical examination. Clinicians should inquire about patient comorbidities, risk factors for RCC (smoking, hypertension, obesity, diabetes), and performance status (3). Serum creatinine and urine dipstick may be utilized to determine chronic kidney disease (CKD) stages (3). Cross-sectional imaging, either computed tomography (CT) or magnetic resonance imaging (MRI), should be obtained, if not already done (3).

Treatment selection for localized RCC can be nuanced and may be influenced by patient, provider, and tumor characteristics (5, 8). Decision aids or risk calculators incorporating patient and tumor characteristics may be used cautiously to personalize counseling and assess morbidity and mortality risk of each treatment option (5, 6). For example, Psutka et al. devised a novel risk calculator which considered patient age, sex, body mass index, CKD stage, ECOG performance status, Charlson comorbidity index, and renal mass diameter to determine the probability of complication or death for RN, PN, ablation, active surveillance, and other causes. Clinicians should engage in shared decision-making with their patients, discussing their desire for invasive treatment and goals for oncologic control, nephron preservation, and minimizing treatment morbidity. Providers should guide patients in carefully balancing the risks and benefits of each treatment option (5, 6).

Ablative therapies may be offered to a variety of patients with SRMs or localized RCC after considering specific patient, tumor, and provider characteristics (5–7). Ideal candidates for ablation include those of older age, with poor performance status, numerous comorbidities, compromised renal function, or a high risk of morbidity from surgical extirpation but are unwilling to accept surveillance (5, 7). Tumors amenable to ablation are small (cT1a masses) and located posteriorly (5, 7). Finally, providers offering ablative therapies should be doing so at a medical center with experience in ablative techniques and access to multidisciplinary care (i.e., interventional radiology, nephrology, medical oncology, etc.) (5). Also, patients who do not desire surgical intervention or want to avoid general anesthesia may be considered for renal mass ablation. For patients selecting ablation, renal mass biopsy should be performed to assist in risk stratification and guide follow-up surveillance (12, 13).

### Guideline recommendations

According to the American Urological Association (AUA), thermal ablation, via either radiofrequency or cryoablation, can be considered for treatment of SRMs <3 cm in size (Table 1) (12). If pursuing thermal ablation, a percutaneous procedure is preferred over the laparoscopic approach. Prior to undergoing ablative therapy, clinicians should counsel patients regarding the increased risk of local recurrence or tumor persistence compared to conventional operative intervention. Patients should also undergo a renal mass biopsy to direct management post intervention (12, 13).

**Table 1:** Recent professional urological society guidelines on the use of focal therapy in localized renal cell carcinoma.

Society	Year	Recommendation
American Society of Clinical Oncology (ASCO)	2017	Percutaneous thermal ablation should be considered an option if complete ablation can reliably be achieved.^a^
American Urological Association (AUA)	2021	Physicians should consider thermal ablation for cT1a renal masses < 3 cm.^b^
European Association of Urology (EAU)	2021	Offer active surveillance or thermal ablation (TA) to frail and/or comorbid patients with small renal masses. Do not routinely offer TA for tumors > 3 cm and cryoablation for tumors > 4 cm.^c^
National Comprehensive Care Network (NCCN)	2022	Thermal ablation is an option for patients with cT1 disease, but may be associated with higher rates of recurrence or persistence in tumors > 3 cm.^d^

a Level of evidence – moderate; ^b^Level of evidence – conditional recommendation, grade C; ^c^Level of evidence – weak; ^d^Level of evidence – 2A.

These guidelines align with those of the European Association of Urology (EAU), which recommend offering thermal ablation as an alternative to surgery for frail and comorbid patients with SRMs. However, the EAU does not explicitly recommend ablative therapies for any other group, and rather states that thermal ablation and cryoablation should not be routinely utilized for tumors less than 3 and 4 cm, respectively. Like the AUA, the EAU also mentions the importance of preablation renal mass biopsy and proper counseling regarding oncologic and procedural risks (14).

To date, both societies acknowledge their recommendations stem from somewhat low-quality evidence with limited follow-up (12–14).

### Ablation modalities

Various ablation modalities, including cryoablation, radiofrequency ablation (RFA), microwave ablation (MWA), and stereotactic ablative radiotherapy (SABR), are currently employed in the treatment of localized RCC. Each method may be performed either percutaneously with image guidance or laparoscopically with direct visualization. Here we review the mechanism of action for each ablative strategy as well as outcomes and current societal recommendations.

## Cryoablation

### Proposed mechanism

**Figure 1:**
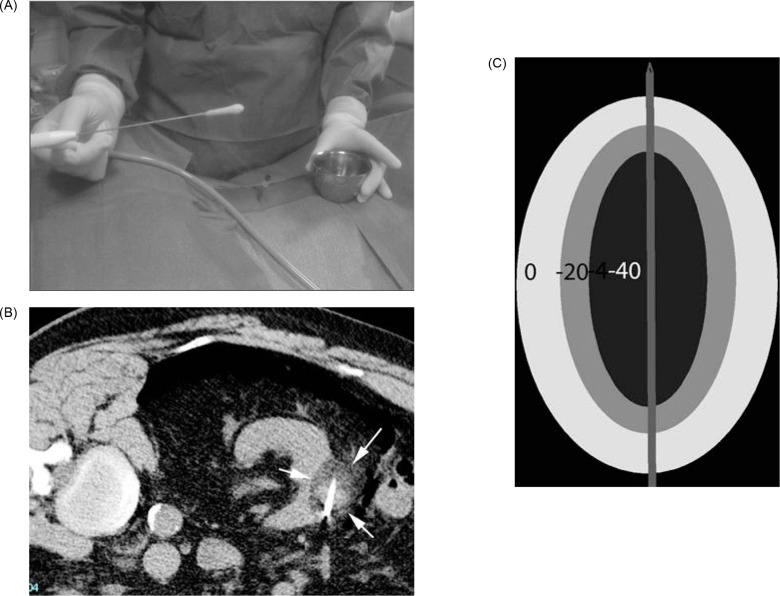
(A) Ice ball seen on the end of cryoablation probe. (B) Ice ball seen in renal tumor during cryoablation. (C) Depiction of isotherms of ice ball.

In cryoablation, the tumor is cooled to a temperature sufficient to obtain cell necrosis. Cryogen gas (typically argon) is depressurized, causing a decrease in temperature at the tip of an antenna—a phenomenon termed the Joule–Thomson effect. Through the tip of the antenna, passive thermal diffusion acts on tumor cells. Intracellular and extracellular crystals are produced by slow and fast freezing cycles (generally two), and these cycles cause cell death through cellular dehydration, vascular thrombosis, and membrane rupture (15). While −20°C is the proven temperature at which cell necrosis occurs in cryoablation, −40°C is the typical temperature achieved in real world practice. The target temperature is obtained at least 3.1 mm inside the ice ball, with shorter distances insufficient for achieving cell death (16) (Figures 1A–1C).

### Outcomes

The literature of cryoablation for the management of SRMs is limited. In this section, we highlight the particular studies that have large sample sizes and or sufficient long-term follow-up. Breen et al. performed a retrospective single-institution analysis regarding efficacy of image-guided cryoablation of T1 renal masses. Major complications (Clavien–Dindo grade ≥ III) occurred in 4.9% (23 out of 473) procedures. Of all 433 patients with T1 renal masses OS was reported as 91.7% (95% CI: 87.5%, 94.5%) and 78.8% (95% CI: 71.1%, 84.6%) at 3 and 5 years, respectively. Recurrence-free survival (RFS) and metastasis-free survival (MFS) were evaluated in a subset of 220 patients with sporadic biopsy-proven RCC. At 3 years, local RFS and MFS were reported as 97.2% (95% CI: 92.6%, 99.0%) and 97.7% (95% CI: 93.3%, 99.1%), respectively. At 5 years, RFS and MFS were calculated at 93.9% (95% CI: 85.8%, 97.4%) and 94.4% (95% CI: 86.7%, 97.7%), respectively (17). Similarly, Pickersgill et al. conducted a single-institution retrospective analysis from 2005 to 2015 of percutaneous cryoablation (PCA) for SRMs to study long-term oncologic outcomes and factors predicting disease recurrence. This study included 308 patients with a mean tumor size of 2.7 ± 1.3 cm. Disease progression rates at a mean follow-up of 38 months were 10.1 and 6.2%, for local recurrence and new lymphadenopathy or metastasis, respectively. After excluding patients with a solitary kidney or Von Hippel–Lindau syndrome, local recurrence and new lymphadenopathy or metastasis occurred in 8.6 and 1.9% of cases, respectively. Disease-free survival (DFS) of PCA was estimated to be 92.5% at 1 year, 89.3% at 2 years, and 86.7% at 3 years. The risk of disease progression increased by 32% with every 1 cm increase in tumor size (18). Additional outcomes of recent observational studies investigating PCA are described in Table 2. Taken together, these studies suggest that cryoablation of SRMs have favorable oncologic outcomes in carefully selected patients. Tumor size can best predict those who will have disease recurrence (17, 18).

**Table 2:** Recent observational studies investigating the role of percutaneous cryoablation in the treatment of localized renal cell carcinoma.

Study	Year	Intervention	Experimental design	Patient population	Number of patients	Outcomes	Media and follow-up	Conclusions
Henderickx et al.	2020	PCA	Retrospective Single institution	Clinical T1 RCC	165	OS DFS RFS	60 months	5-yr OS = 74.0% 5-yr DFS = 96.9% 5-yr RFS = 95.4%
Bhagavatula et al.	2020	PCA	Retrospective Single institution	Biopsy-proven, T1 RCC	307	OS DSS DFS LPFS	95 months	10-yr OS = 78% 10-yr DSS = 99% 10-yr DFS = 88% 10-yr LPFS = 95%
Morkos et al.	2020	PCA	Retrospective Propensity-matched	Biopsy-proven, Stage 1 RCC	134	OS RFS DSS	88.8 months	5-yr OS = 87% 5-yr DSS = 94% 10-yr OS = 72%
Gobara et al.	2021	PCA	Prospective Single institution	Biopsy-proven, T1a RCC	33	CSS OS	60.1 months	5-yr CSS = 100%5-yr OS = 96.8%

CSS, cancer-specific survival; DFS, disease-free survival; DSS, disease-specific survival; LPFS, local progression–free survival; OS, overall survival; PCA, percutaneous cryoablation; RCC, renal cell carcinoma; RFS, recurrence-free survival.

Several groups have also reported on the difference in laparoscopic and PCA. Kim et. al compared PCA in 123 tumors with laparoscopic cryoablation (LCA) in 167 tumors. Both groups shared no difference in decline in glomerular filtration rate (GFR) or complication rates. In terms of oncologic outcomes, 5-year OS and RFS rates were 86.3 and 86.3%, respectively, for PCA, and 79.3 and 85.5%, respectively, for LCA. Multivariate Cox proportional hazards analysis demonstrated that cryoablation approach regardless of mechanism was not predictive of overall mortality or disease recurrence (P = 0.36 and 0.82, respectively), concluding that oncologic outcomes were not fully attributed to cryoablation approach (19). Other authors have corroborated these findings as well (20).

To date, there are currently two registered clinical trials studying cryoablative techniques for RCC. NCT04506671 is a prospective, nonrandomized trial comparing local recurrence in patients with T1b renal tumor receiving cryoablation or PN. The primary outcome is local recurrence for up to 5 years, while secondary outcomes include metastatic progression, quality of life, renal function, rate of adverse events, blood loss, length of stay, and pain scores (21). NCT04040530 is a prospective cohort study comparing patients with T1 biopsy-proven RCC who undergo treatment with PN or cryoablation. Main outcomes include change in quality of life, rehabilitation from treatment, complication and readmission rates, and treatment success after 3 months (22). These studies will hopefully inform how to best include cryoablation in our armamentarium for treating SRMs. Cryoablation in contrast to radiofrequency and MWA does not have a coagulative effect theoretically leading to increased bleeding complications, although this has not been clinically relevant. In addition, unlike microwave and RFA, cryoablation does not destroy collagen, resulting in less long-term injury to the collecting system. As with all ablative therapies long-term, active follow-up is essential.

### Guideline recommendations

AUA guidelines acknowledge cryoablation as an option for patients who select ablative therapy instead of surgery as treatment for their renal mass. They recognize that there is no significant difference between cryoablation and RFA in complications, metastatic progression, or CSS (12, 13). The American Society of Clinical Oncology (ASCO) similarly recommend, “percutaneous thermal ablation should be considered an option for patients who possess tumors such that complete ablation will be achieved” (23).

## Radiofrequency Ablation

### Mechanism

**Figure 2:**
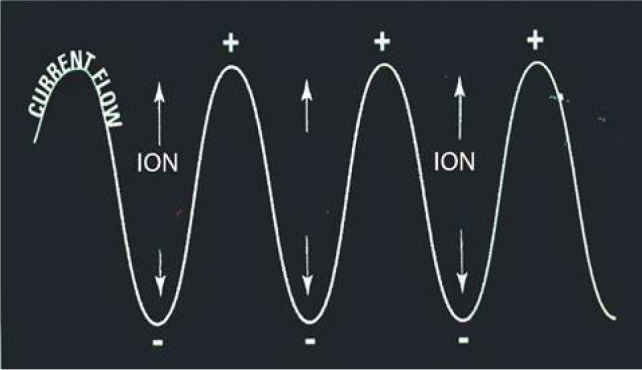
Depiction of radiofrequency causing ionic agitation and subsequent frictional heating.

**Figure 3:**
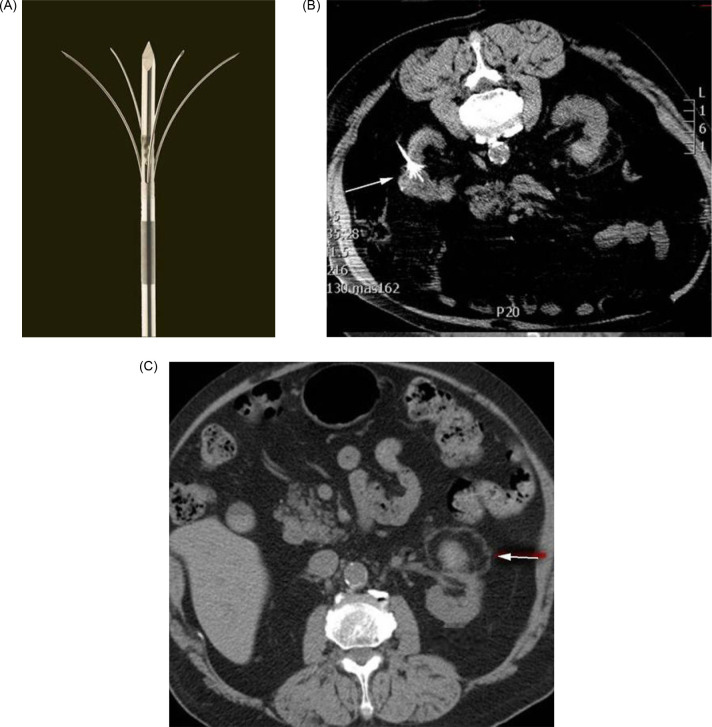
(A) Multi-tined radiofrequency ablation antenna. (B) Radiofrequency ablation antenna expanded within tumor. (C) Typical tissue changes after successful radiofrequency ablation including pseudo-capsule, subcapsular fat, and nonenhancing lesion.

Radiofrequency ablation uses an electrical current in the radio frequency range, between 3 Hz and 300 GHz, to heat tissue resulting in coagulative necrosis. The alternating current is delivered through antennas placed directly into the tumor, with the electrical circuit completed through grounding pads, such as those seen with the Bovie cautery device (24). As current passes through the tissue, heat is generated due to tissue resistance, a concept termed “resistive heating” (Figure 2) (25). Cell death occurs instantly at temperatures above 60°. Notably, effective delivery of RFA relies on the electrical and thermal conductivity in tissue, which can be adversely affected by overheating and desiccation of the tissue directly adjacent to the electrode, resulting in an insulating barrier of charred tissue (26). RFA is often not recommended adjacent to large vasculature, due to inability to overcome the heat sink effect leading to increased risk of incomplete necrosis, possibly impacting oncologic efficacy (27). Over the years, a variety of techniques have been developed to monitor tissue temperature and impedance to limit desiccation and increase the zone of ablation (28). Similarly, multi-tined, expandable, and perfusion electrodes, and bipolar electrode systems are available technologies used to modulate the size and shape of the ablated tissue (26) (Figures 3A–3C).

### Outcomes

Most data supporting the role of RFA is retrospective in nature and suggests that RFA is feasible in most instances (Table 3). A study by Zhou et al. explored the therapeutic and renal function outcomes of 297 patients who underwent image-guided percutaneous ablation of T1a biopsy-proven RCC between 2006 and 2016 (29). Of their cohort of 297 patients, 244 (82%) of patients underwent RFA. Technical success, defined as the ability to successfully perform an ablative procedure, was achieved in 100% of patients, with a 16% rate of adverse events. At 1-month, primary efficacy, defined as the ability to successfully treat a tumor with one ablative session, was achieved in 233 of 244 patients (95%), with secondary efficacy after repeat ablation achieved in 100% of patients. During their follow-up of 2 years, there were no reported instances of local recurrence. With regard to renal function, they found no difference between preablation and postablation GFR (29).

**Table 3:** Recent publications investigating oncologic outcomes in patients undergoing RFA for localized renal carcinoma.

Study	Year	Experimental design	Intervention	Patient population	Number of patients	Outcomes	Mean/Median follow-up	Conclusions
Clark et al.	2007	Phase I clinical trial	MWA	Solid, enhancing renal mass on imaging No size limitations	Total = 10	Ablation size Presence of skip areas	Not reported	Single probe ablation size = 4.1 × 2.7 × 2.2 cm Triple probe ablation size = 5.7 × 4.7 × 3.8 cm No skip areas in any patient
Liang et al.	2008	Phase I clinical trial	MWA	Biopsy proven cT1a RCC	Total = 12	Feasibility Safety Efficacy	Median = 11 months	No complications 100% technical success rate
Bartoletti et al.	2012	Phase I clinical trial Multi-institution	MWA	Solid, enhancing renal mass on imaging Candidate for open radical nephrectomy	Total = 14	Coagulation parameters Bleeding Ablation size	Mean = 27.4 months	No significant effects of coagulation No local bleeding after MWA Mean ablation size = 44.14 mm (±22.59 mm)
Guan et al.	2012	RCT	MWA	Solitary, unilateral, cT1a	Total = 102 MWA = 48 PN = 54	RFS	Median MWA = 32 months Median PN = 36 months	In the pathologically confirmed subgroup =3-yr RFS: 90.4% vs 96.6%; P = 0.465
Yu et al.	2015	Retrospective Single institution	MWA vs RN	Biopsy-proven T1a RCC	Total = 426 MWA = 98 RN = 328	OS CSS	Median MWA = 25.8 months Median RN = 26.1 months	5-yr OS = 82.6 vs 98.6%; P = 0.0004 5-yr CSS = 97 vs 98%; P = 0.38
Vanden Berg	2021	Retrospective Single institution	MWA	Biopsy-proven RCC (including oncocytic)	Total = 101 Biopsy-proven RCC = 82	RFS	Median = 12.4 months	40 months, RFS in biopsy-proven RCC = 93.3%
Yu et al.	2020	Retrospective Propensity matched	MWA vs PN	Biopsy-proven T1a RCC	Total = 1955 MWA = 185 PN = 1770	LTP CSS Metastasis	Median = 40.6 months	LTP = 3.2% vs 0.5%; P = 0.10 CSS = 2.2% vs 3.8%; P = 0.24 Metastases = 4.3% vs 4.3%; P = 0.76
Yu et al.	2022	Retrospective Multi-institution	MWA	Biopsy-proven T1 RCC	Total = 323 cT1a = 275 cT1b = 48	LTP DFS CSS OS	cT1a Median = 66 months cT1b Median =30.4 months	10-yr OS = 67.5% 10-yr DFS = 71.8% 10-yr CSS = 87.4% 10-yr LTP = 1.9% 5-yr OS = 89.2% 5-yr DFS = 69.1% 5-yr CSS = 91.4% 5-yr LTP = 11.3%

CSS, cancer-specific survival; DFS, disease-free survival; LTP, local tumor progression; MWA, microwave ablation; OS, overall survival; RCC, renal cell carcinoma; RFS, recurrence-free survival; PN, partial nephrectomy; RN, radical nephrectomy.

Data regarding oncologic outcomes for RFA are promising. Olweny et al. retrospectively compared the oncologic outcomes of RFA to PN, the current gold standard of localized RCC (30). Despite increased age and comorbidity burden, there was no difference in OS in RFA vs PN at 5 years (97.2% vs 100%; P = 0.31) in their cohort of biopsy-proven RCC patients. Neither age, tumor size, histology nor duration of follow-up was a significant predictor of oncologic outcomes. Another long-term study by Psutka et al. concluded that RFA can result in durable local control with low risk of recurrence (10). Johnson et al. also explored long-term outcomes in patients who underwent RFA and found that DFS and CSS were 89 and 96%, respectively, at 6 years for those with tumors less than 3 cm. However, in individuals with tumors greater than 3 centimeters, DFS decreased significantly to 68% (31). In their sub-group analysis of individuals with biopsy- proven RCC with at least 10-years of follow-up, MFS and CSS were both 94%. Similar, recent, retrospective studies have shown comparable long-term onco-logic outcomes in those undergoing RFA vs PN (32–34).

However, despite the encouraging body of literature, RCTs are needed to clarify the role of RFA. Unfortunately, there are no published RCTs’ data comparing RFA to PN for SRMs. However, NCT00019955, a phase II trial assessing the feasibility of RFA in slowing destroying tumor tissue and preventing tumor growth, has completed enrollment. No results have been posted to date (35).

### Guideline recommendations

**Figure 4:**
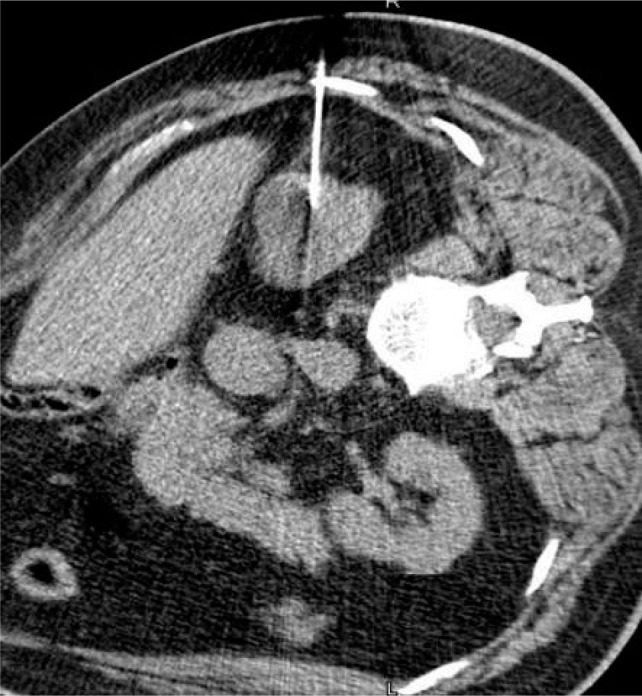
(A) Microwave antenna seen in 3 cm, upper pole renal mass. (B) Follow-up at 3 months shows residual enhancement of the superior aspect of the lesion consistent with residual tumor, demonstrating the importance of follow-up.

Strong retrospective results in the setting of a lack of available randomized trial data have resulted in relatively broad guideline recommendations for the use of RFA. The AUA notes that thermal ablation, “should be considered as an alternative approach for the management of cT1a renal masses < 3 cm”(12, 13). The EAU states that thermal ablation can be offered, “to frail and/or comorbid patients with SRMs.” They caution that, “low quality studies suggest high disease recurrence rates after RFA of tumors > 3 cm (14) (Figures 4A and 4B).

## Microwave Ablation

### Mechanism

**Figure 5:**
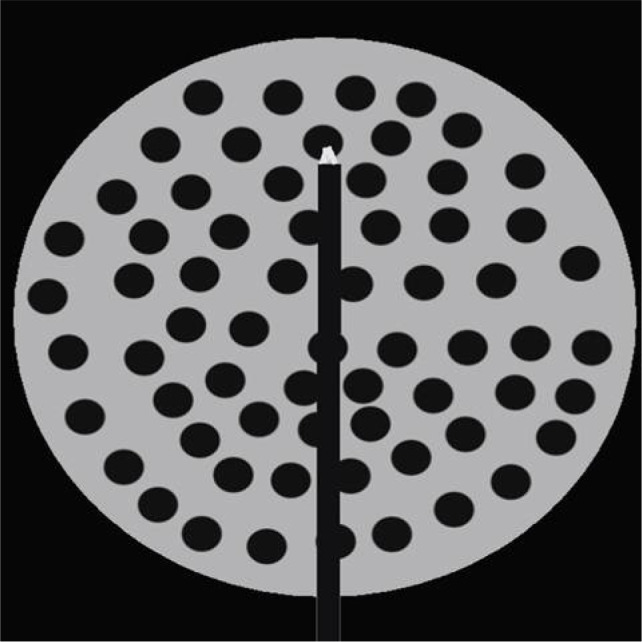
Depiction of microwave antenna generating a field of heat.

Microwave ablation is a form of electromagnetic radiation that typically oscillates between 900–2500 MHz (36). Like RFA, this fluctuation causes the continued realignment of polar molecules within tissue producing an increase in kinetic energy. This phenomenon, termed “dielectric hysteresis,” translates to rising temperature within the tissue, often above 100°C (15, 36). In MWA, these electromagnetic waves are delivered through one or more antennae inserted into the tissue (27). In contrast to RFA, MWA does not rely on tissue conductivity, and can effectively heat a variety of tissues regardless of electrical conductivity (37). This field effect provides for uniform heat generation (Figure 5). Thus, it is effective compared to RFA for lung, bone, and other tissues with high electrical impedance (37). Further, MWA has an increased ability to overcome the heat sink effect (38). This is an advantage for efficacy within highly vascular organs such as the kidneys, where increased blood flow can lead to the dispersion of thermal energy (27).

### Outcomes

There is limited data regarding the efficacy of MWA. In an early study by Liang et al. that evaluated the feasibility, safety, and efficacy of MWA for RCC, a total of 12 patients with biopsy-proven RCC from 1.3 cm to 3.8 cm in diameter underwent MWA (Table 4) (39). All tumors were completely ablated with a single session of MWA. No complications were reported, and no recurrence was observed at a median follow-up of 11 months. Similarly, two phase I studies, with fewer than 15 patients who underwent MWA prior to nephrectomy, concluded that MWA can safely and quickly ablate renal tumors (40, 41).

**Table 4:** Recent publications evaluating efficacy and safety of microwave ablation for the treatment of localized renal cell carcinoma.

Study	Year	Experimental design	Intervention	Patient population	Number of patients	Outcomes	Mean/Median follow-up	Conclusions
Olweny et al.	2012	Retrospective Single institution	RFAvs PN	Biopsy-proven, T1a RCC	RFA cohort = 37 PN cohort = 37	OS CSS RFS DFS MFS	RFA Median = 6.5 years PN Median = 6.1 years	5-yr OS = 97.2 vs 100%; P = 0.31 5-yr CSS = 97.2 vs 100%; P = 0.31 5-yr DFS = 89.2 vs 89.2%; P = 0.78 5-yr RFS = 91.7 vs 94.7%; P = 0.96 5-yr MFS = 97.2 vs 91.5%; P = 0.35
Psutka et al.	2013	Retrospective Single institution	RFA	cT1 RCC	RFA cohort = 185	RFS MFS CSS DFS OS	Median = 6.43 years	5-yr RFS = 95.2% 5-yr MFS =99.4% 5-yr CSS = 99.4% 5-yr DFS =87.6% 5-yr OS = 73.3% 10-yr RFS T1a = 93.2%
Chang et al.	2015	Retrospective Single institution	RFA vs PN	Biopsy-proven, T1b RCC	RFA cohort = 27 PN cohort = 29	OS CSS DFS	RFA Mean = 65.9 months PN Mean = 70.2 months	5-yr OS = 85.5 vs 96.6%; P = 0.14 5-yr CSS = 92.6 vs 96.6; P = 0.49 5-yr DFS = 81.0 vs 89.7%; P = 0.36
Ji et al.	2016	Retrospective Single institution	RFA vs PN	Biopsy-proven, T1a RCC	RFA cohort = 105 PN cohort = 74	OS CSS DFS	RFA = Median 78 months PN = Median 82 months	5-yr OS = 93.3 vs 94.6%; P > 0.05 5-yr CSS = 98.0 vs 98.5; P > 0.05 5-yr DFS = 97.1 vs 97.3%; P > 0.05
Andrews et al.	2019	Retrospective Single institution	RFA vs PN vs PCA	Clinical T1 RCC	RFA cohort = 180 PN cohort = 1055 PCA cohort = 187	CSS	RFA Median = 7.5 years PN Median = 9.4 years Cryo Median = 6.3 years	5-yr CSS cT1a = 95.6 vs 99.3 vs 100% 5-yr CSS cT1b = N/A vs 98% vs 91%
Park et al.	2019	Retrospective Single institution	RFA vs PN	Biopsy-proven, T1a RCC	RFA cohort = 62	OS	RFA Mean = 60 months	5-yr OS = 98.4 vs 100%; P = 0.360
Johnson et al.	2019	Retrospective Single institution	RFA	Contrast enhancing, nonmetastatic renal mass	Total = 106 Biopsy-proven w/ 10-yr f/u = 39	MFS CSS	Median = 79 months	10-yr biopsy proven MFS = 94% 10-yr biopsy proven CSS = 94%
Zhou et al.	2019	Retrospective Single institution	RFA vs PCA vs MWA	Biopsy proven, T1a RCC	RFA cohort = 244 PCA cohort = 26 MWA cohort = 27	DFS MFS CSS	Median = 24 months	2-yr DFS = 100 vs 100 vs 100% 2-yr MFS = 100 vs 100 vs 100% 2-yr CSS = 100 vs 100 vs 100%

CSS, cancer-specific survival; DFS, disease-free survival; MFS, Metastasis-free survival; MWA, microwave ablation; OS, overall survival; PCA, percutaneous cryoablation; PN, partial nephrectomy; RCC, renal cell carcinoma; RFA, radiofrequency ablation; RFS, recurrence-free survival.

More recently, Yu et al. retrospectively compared the efficacy of MWA (n = 98) and laparoscopic RN (n = 328) in patients with RCC ≤ 4 cm. At 5 years, CSS was comparable between MWA and laparoscopic RN (97% vs 98%; P = 0.38) (42). An even larger study by Yu et al. comparing 1955 propensity-matched patients with cT1a RCC undergoing MWA or laparoscopic RN found no difference in local progression (3.2% vs 0.5%, respectively; P = 0.10) and CSS rates (2.2% vs 3.8%, respectively; P = 0.24) (43). MWA was associated with decreased decline in GFR (6.2 vs 16.4%; P < 0.001). However, when compared to laparoscopic RN, MWA was associated with worse OS (HR = 2.4; 95%CI 1.0–5.7; P = 0.049) and DFS (82.9% vs 91.4%; P = 0.003). These findings may be attributed to poor overall health and increased comorbidities within the patient population selected to undergo MWA. The most recent study by Yu et al. further reinforces that MWA is a safe, reliable option for patients with cT1 RCC. After a median follow-up of 66 months for patients with T1a patients, CSS, DFS, and OS rates were 87.4, 71.8, and 67.5%, respectively. For T1b patients with a median follow-up of 30.4 months, CSS, DFS, and OS rates were 91.4, 69.1, and 89.2%, respectively. Interestingly, technical success was achieved in 97% of patients despite over 40% of tumors inhabiting dangerous locations (near bowel or the collecting system) (44).

To date, there has only been a single published prospective, randomized controlled trial investigating MWA vs PN. The authors randomized 102 patients with a renal mass ≤ 4 cm to open PN (n = 19), laparoscopic PN (n = 35), open MWA (n = 20), or laparoscopic MWA (n = 28). At median follow-up of 32 and 36 months for the MWA and PN, respectively, there was no significant difference in local RFS (91.3% vs 96.0%; P = 0.4650 (45). This limited length of follow-up underscores the need for additional, long-term studies to effectively evaluate oncologic outcomes of MWA for SRMs. To our knowledge there are no active, prospective RCTs investigating the role of MWA. While MWA is best reserved for T1a lesion, recent reports have appeared for MWA inT1b lesions. In one such single-center, retrospective study including 23 patients with T1b RCC, primary technical success was achieved in 20/23 (87%) patients (46), and secondary technical success was achieved in 3/3 (100%) patients. Local tumor progression-free survival (PFS) was 100.0, 90.9, and 90.9% at 1, 2, and 3 years, respectively. Overall survival (OS) was 95.2, 85.7, and 71.4% at 1, 2, and 3 years, respectively.

### Guideline recommendations

Current EAU guidelines classify MWA as experimental, but comment that the data seems to support equivalence to RFA and cryoablation in terms of safety and oncologic outcomes over the short term. However, the EAU panel cites inadequate data regarding the clinical efficacy of thermal ablation compared to PN (47). AUA guidelines also classify MWA as investigational, but further clarify that percutaneous ablation techniques can be considered as an alternate approach to manage cT1a masses (12, 13). However, many institutions have adopted MWA as a suitable replacement for RFA.

## Stereotactic Ablative Radiotherapy

### Mechanism

In contrast to laparoscopic or percutaneous ablation techniques, stereotactive body radiotherapy (SBRT) is completely noninvasive, without need for an anesthetic procedure, and is characterized by delivery of high-dose, hypo-fractionated ionizing radiation to the tumor (48). Similar to other ablative therapies, SBRT is associated with low toxicity and is available in the outpatient setting (49).

Historically thought to be radioresistant, the higher dose, hypo-fractionated nature of SBRT has been effective in treating RCC (50), challenging current treatment paradigms. The precise mechanism of SBRT is unclear, but some hypothesize that the antitumor effects are mediated through acid sphingomyelinase, which under the effects of the single-fraction, high-dose radiation characteristic of SBRT translocates to the cell membrane where it catalyzes lysis of sphingomyelin to ceramide, an intracellular messenger known to coordinate proapoptotic signaling (51). Besides from inducing direct cellular damage (52), SBRT may induce expression of MHC I, adhesion molecules, heat shock proteins, and other inflammatory and immunomodulators, resulting in a local tumor response (53). These immunomodulatory effects of SBRT can extend beyond the local irradiated area to affect meta-static sites (54). In 1953, this phenomenon was termed the “abscopal effect” and has been reported in numerous types of malignancies including RCC (54, 55).

### Outcomes

While SBRT has historically had a role in the treatment of lung, liver, and bone tumors, it is becoming increasingly important in the treatment of RCC (51). To evaluate the role of SBRT in primary RCC, Correa et al. performed a meta-analysis including 26 studies and 372 patients, 80% (n = 300) of whom had localized disease (Table 5) (56). The study estimated a local control rate of 97.2% (95% CI 93.9– 99.5%) for the entire cohort, a finding the authors note to be comparable to thermal ablation, when considering tumor size. Furthermore, Yamamoto et al. investigated long-term oncologic outcomes in clinical or recurrent T1 RCC patients who underwent SBRT. In their cohort of 29 patients, 5-year localized control (LC), loco-regional control (LRC), PFS, DFS, and OS were 94, 88, 50, 96, and 68%, respectively (57). In the nonmetastatic, T1b RCC population treated with SBRT, Siva et al. observed a 4-year CSS, OS, and PFS of 91.4, 69.2, and 64.9%, respectively. However, OS and PFS are dominated by death from other causes in this cohort. Local, distance, and any failure at 4 years were 2.9, 11.1, and 12.1%, respectively (58).

**Table 5:** Recent retrospective studies on the oncologic outcomes of stereotactic body radiotherapy to treat localized renal cell carcinoma.

Study	Year	Experimental design	Intervention	Patient population	Number of patients/studies	Outcomes	Follow-up	Conclusion
Correa et al.	2019	Systematic review, meta-analysis	SBRT	All stages, RCC	Studies = 26 Patients = 372	LC	Median = 28 months	LC = 97.2%
Siva et al.	2020	Retrospective Multi-institution	SBRT	≥ T1b, nonmetastatic RCC	Patients = 95	CSS OS PFS	Median = 2.7 years	4-yr CSS = 91.4% 4-yr OS = 69.2% 4-yr PFS = 64.9%
Yamamoto et al.	2021	Retrospective Single institution	SBRT	Clinical or recurrent T1 RCC	Patients = 29	LC LR CPF DSS OS	Median = 57 months	5-yr LC = 94% 5-yr LRC = 88% 5-yr PFS = 50% 5-yr DSS = 96% 5-yr OS = 68%

CSS, cancer-specific survival; DSS, disease-specific survival; LC, local control; LRC, loco-regional control; OS, overall survival; PFS, progression-free survival; RCC, renal cell carcinoma; SBRT, stereotactive body radiotherapy.

This encouraging retrospective data has spurred interest in prospective clinical studies (Table 6). One such study, FAS-TRACK (NCT01676428), recruited 37 patients with T1a (n = 13), T1b (n = 23), and T2a (n = 1) RCC (59). Distant progression-free survival was 97% (95% CI: 91–100%) at 1 year and 89% (95% CI: 78–100%) at 2 years. These results led to the development of FASTRACK II (NCT02613819), a phase II, single-arm, interventional trial investigating the role of SBRT in patients with biopsy-confirmed RCC ≤ 8 cm who were medically inoperable, at high risk, or were declined surgery (60). Like FASTRACK II, AQuOS-RCC (NCT03108703) and NCT03811665 are active, non-recruiting phase I studies (61, 62). AQuOS-RCC primarily aims to assess the quality of life in patients undergoing SBRT for primary renal lesions ≥ 2.5 cm or recurrent lesions following local ablative therapy. In addition, there are two phase II trials examining SBRT in the setting of localized RCC: AQuOS-II NCT05023265 and NCT02141919 (63, 64). Notably, there are two actively recruiting trials, NCT01890590 and NCT04115254, studying the use of Cyberknife® and stereotactic magnetic resonance–guided radiation therapy (SMART) in the setting of Stage 1 RCC (65, 66).

**Table 6:** Prospective interventional clinical trials evaluating the oncologic outcomes of SBRT and similar radiotherapy for the treatment of localized renal cell carcinoma.

Study	Status	Experimental design	Intervention	Patient population	Patient enrollment	Outcomes	Follow-up	Conclusions
FASTRACK NCT01676428	Complete	Phase I Single-arm Interventional trial	SBRT	T1a–T2a RCC and Medically inoperable or High risk for surgery or Decline surgery	Final Enrollment = 37	AE LPFS DPFS OS	Median = 24 months	Grades 1 & 2 toxicity = 78% Grade 3-4 toxicity = 3% Grades 4 & 5 toxicity = 0% 2-yr LPFS = 100% 2-yr DPFS = 89% 2-yr OS = 92%
FASTRACK II NCT02613819	Active, not recruiting	Phase II Single-arm Interventional trial	SBRT	Biopsy-confirmed RCC ≤ 8 cm and Medically inoperable or High risk or Decline surgery	Current Enrollment = 71	PFS	Ongoing	Ongoing
AQuOS-RCC NCT03108703	Active, not recruiting	Phase I Single-arm Interventional trial	SBRT	Lesion ≥ 2.5 cm or Recurrent lesion following local ablative therapy	Enrollment Goal = 30	QOL LC PFS OS AE	Ongoing	Ongoing
AQuOS-II NCT05023265	Not yet recruiting	Phase II Single-arm Interventional trial	SBRT	Primary lesion 3–20 cm and Medically inoperable or Decline surgery	Enrollment Goal = 46	LC PFS OS QOL AE	Ongoing	Ongoing
NCT01890590	Recruiting	Phase II Single-arm Interventional trial	Cyberknife	Stage I RCC	Enrollment Goal = 46	LPFS QOLAE	Ongoing	Ongoing
NCT03811665	Active, not recruiting	Phase I Two-arm Interventional trial	SBRT vs RFA	Biopsy-confirmed T1a RCC	Enrollment Goal = 24	Treatment failure OSDFSRFS	Ongoing	Ongoing
NCT04115254	Recruiting	Phase I-II Parallel arms Interventional trial	SMART	Confirmed malignancy and Tumor ≤ 7 cm	Enrollment Goal = 1000	Delivery success Tumor visualization Plan creation Tumor control	Ongoing	Ongoing
NCT02141919	Active, not recruiting	Phase II Single-arm Interventional trial	SBRT	Biopsy-proven RCC (including oncocytoma) and Tumor ≤ 5 cm	Current Enrollment = 16	Tumor growth AE Renal function PFS	Ongoing	Ongoing

AE, adverse events; DFS, disease-free survival; DPFS, disease progression–free survival; LC, local control; LPFS, local progression–free survival;OS, overall survival; PFS, progression-free survival; QOL, quality of life; RCC, renal cell carcinoma; RFA, radiofrequency ablation; RFS, recurrence-free survival; SBRT, stereotactic body radiotherapy; SMART, stereotactic magnetic resonance–guided radiation therapy.

SBRT treatment is exceptionally well tolerated. In a prospective dose-escalation trial, the only acute toxicities were Grade I fatigue (45%) and Grade I nausea (11%). Among the 11 patients, there were two late complications—one Grade 2 with decline in renal function and one Grade 3 with episode of pyelonephritis (67). Similar low toxicity rates were reported by Siva et al. (58).

### Guideline recommendations

Current EAU guidelines comment that while stereotactic radiotherapy results are encouraging, additional randomized control data are required to clarify the role in the management of localized RCC (47). AUA guidelines similarly categorize SBRT as investigational, remarking it should be considered for patients who are both medically inoperable and not candidates for traditional thermal ablative modalities (12, 13).

## Failure Rates

Although recent studies of focal therapy for the treatment of localized RCC have been promising, a proportion of patients require reintervention due to recurrence or an incomplete eradication of the primary tumor. In a study investigating the long-term oncologic outcomes in patients with T1 RCC, Psutka et al. noted that 13% (n = 24) of patients required retreatment for residual disease (10). Further, 6.5% (n = 12) of patients in their study experienced local recurrence. Small but notable local tumor progression has been observed in those treated with MWA, with Yu et al. showing 5-year local tumor progression rates of 1.9 and 11.3% for patients with cT1a and cT1b RCC, respectively (44). In the context of cryoablation, Bhagavatula et al. reported evidence of recur-rent RCC on imaging after initial treatment in 7.9% (n = 23) of patients. Notably, local progression occurred in 4.5% (n = 13) with 1.0% (n = 3) of patients unfortunately developing metastatic disease (68). In their meta-analysis investigating SBRT, Correa et al. identified a local control rate of 92.2%, but included studies with local controls rates from 70–100%. They clarify that local failure tended to occur in those treated with low-dose SBRT, a finding that has been seen in other malignancies treated with SBRT (56).

It is vital to place these local failure rates in the context of PN. Data comparing ablation with PN are largely retrospective and somewhat heterogeneous. Some studies support worse oncologic results with ablation. For example, Fraisse et al. used radiological evidence and the RENAL nephrometry score to pair similar patients treated by PCA and robotic-assisted partial nephrectomy (RAPN). In their study of 647 patients, the absolute recurrence rates were 2.8% in the RAPN cohort versus 8.4% in the PCA cohort (P = 0.03) (69). However, other studies report more comparable onco-logic outcomes between ablative and surgical therapies. Bianchi et al. associated PN with higher DFS in comparison with thermal ablation (92.8% vs 80.4%, respectively; P = 0.02) but no difference in OS between either treatment modality (70). Andrews et al. reported on 1422 patients withT1a RCC and demonstrated a 5-year CSS to be 99, 96, and 100% for PN, RFA, and cryoablation, respectively (9).

Moving forward, prospective, randomized studies will be critical in accurately assessing local failure rates and discerning oncologic outcomes between ablation and PN. Efforts to refine focal therapy techniques to minimize positive margins and incomplete treatment are also crucial (71).

## Future Perspectives

Undoubtedly, it is an exciting time for those involved in the treatment of RCC with the rapid expansion of treatment modalities. In addition to thermal ablation, SABR, and MWA monotherapy, there is a recent, growing interest in combination therapies. In a small series of seven patients with an average tumor size of 6.4 cm, Blitzer et al. used a combination of MWA and SBRT to achieve local control rates of 100% at a median follow-up of 15 months (72).

Novel technologies continue to be utilized for the treatment of RCC. While proton beam therapy has an established role in other malignancies, clinicians are beginning to explore its application in the treatment of RCC. In a multi-institutional study of 22 patients with a median follow-up of 37 months, Fukumitsu et al. reported 3-year OS and 3-year CSS rates of 950 and 100%, respectively, for primary RCC patient undergoing proton therapy. No major complications or effects on serum blood urea, nitrogen, or creatinine were reported (73). Another emerging treatment modality for SRMs is irreversible electroporation (IRE). This technology delivers a pulsed electrical field resulting in irreversible permeabilization of tumor cell membranes (74). Early studies by Wah et al. and Dai et al. have shown promising results with local RFS rates of 91% at 3 years and 81% at 5 years, respectively (75, 76). Additional larger studies of combination therapies and novel therapeutics alongside RFA, PCA, SABR, and MWA are necessary to discern the best ablative therapy options for SRM treatment.

## Conclusion

The growing incidence of SRMs and an aging population has increased the demand for novel, nonsurgical therapies. Professional societies are increasingly embracing ablative therapies for treatment of SRMs, particularly in poor surgical candidates or those who do not desire surveillance. RCTs and maturation of long-term oncologic outcomes of up-and-coming technologies such as MWA, IRE, SABR, and others are needed to further expand the armamentarium of urologic oncologists, interventional radiologists, and radiation oncologists.
